# *Burkholderia gladioli* strain NGJ1 deploys a prophage tail-like protein for mycophagy

**DOI:** 10.15698/mic2018.02.617

**Published:** 2017-12-31

**Authors:** Rahul Kumar, Sunil Kumar Yadav, Durga M. Swain, Gopaljee Jha

**Affiliations:** 1 Plant Microbe Interactions Laboratory, National Institute of Plant Genome Research, Aruna Asaf Ali Marg, New Delhi-110067, India.

**Keywords:** bacterial mycophagy, phage tail protein, sheath blight disease, Fungal diseases, rice, microbiome, anti-fungal compound, biocontrol

## Abstract

Fungal pathogens are responsible for approximately two third of the infectious plant diseases. Historically they have been associated with several devastating famines, causing death and disabilities in humans. Mostly fungal diseases are being controlled by using fungicides which otherwise have adverse side effects on the health of consumers as well as environment. Due to extensive usages, pathogens have evolved resistance against most of the commonly used fungicides and rendered them ineffective. Controlling fungal disease in a sustainable and eco-friendly fashion remains a challenge. The antifungal biocontrol agents are being considered as potent, alternative and ecofriendly approach to manage fungal diseases. In our recent work, we have identified a rice associated bacterium; *Burkholderia gladioli* strain NGJ1 which demonstrates broad spectrum fungal eating (mycophagous) property. We determined that the bacterium utilizes its type III secretion system (Injectisome) machinery to deploy a prophage tail-like protein (Bg_9562) into fungal cells to devour them. The purified Bg_9562 protein from over-expressing recombinant *E. coli *strain demonstrates broad spectrum antifungal activity. Overall our study opens up a new opportunity to exploit prophage tail-like protein as potent antifungal compound to control plant as well as animal fungal diseases.

Rice is a staple food crop, feeding more than half of the entire human population of the world. However, it is susceptible to several diseases which pose a serious threat for sustainable rice cultivation. Amongst them, the sheath blight disease (SBD) caused by a necrotrophic fungal pathogen *Rhizoctonia solani* is known to affect 6-69% of rice cultivation. Due to lack of natural source of complete disease resistance, the disease is mostly controlled by usages of fungicides. While exploring various strategies to control SBD, we isolated a rice associated yellow pigmented bacterium and found it efficient in controlling the growth of *R. solani *in laboratory condition. Upon establishing the pure culture, through rDNA sequencing as well as draft genome sequence analysis, we identified the bacterium as *Burkholderia gladioli*. The strain was named as NGJ1, as it was isolated at **N**IPGR, New Delhi by us (**G**opaljee **J**ha, being lead researcher). It is fascinating that the bacterium was found growing over the fungal biomass, in a week old confrontation plates. In general, the antifungal bacteria develop inhibitory zones, but we observed that this bacterium can cross the inhibitory zone and spread over fungal biomass. Only limited numbers of secondary sclerotia were formed on such plates and they were non-viable. Also the fungal mycelia collected from confrontation plates were disintegrated, cytoplasm squeezed and nuclei being fragmented. Overall it suggested that the NGJ1 could disintegrate living fungal biomass and induce cell death responses. We reinforced these observations by treating the pre-grown fungal biomass with bacterial cultures in liquid broth or on glass slides having thin layer of agar. The MTT, trypan blue and PI staining suggested the bacterial treated mycelia to be dead. In addition, we observed reduction in fungal biomass in bacterial treated samples. This suggested that during confrontation, the NGJ1 not only kills fungi but also degrades them to potentially utilize them as nutrient source. Indeed we observed leakage of fungal metabolites/cytoplasmic granules from the disintegrating mycelia during microscopic analysis. Also drastic increase in bacterial growth was observed in minimal media in presence of fungal mycelia.

Upon successfully demonstrating that the NGJ1 exhibits mycophagous ability on* R. solani*, we further explored whether the bacterium demonstrates such property on other fungi. It was observed that this bacterium is capable of feeding on several different strains of *R. solani* as well as other important fungal pathogens, including *Fusarium oxysporum*, *Magnaporthe oryzae*, *Venturia inaequalis*, *Ascochyta rabiei*, *Alternaria brassicae and Candida albicans *(a human pathogen). Generally the fungi has chitinaceous cell wall, we reasoned that somehow the NGJ1 is capable of sensing this conserved feature to recognize fungi to feed on them. However we observed that the bacterium can also forage over a *Phytophthora* sp., an oomycete pathogen having non-chitinaceous cell wall. Thus it is still intriguing how the NGJ1 could recognize broad range of fungi to exhibit mycophagous ability.

Upon establishing that the NGJ1 shows mycophagous activity, we got interested in exploring its molecular mechanism. First of all, we observed that a functional type III secretion system (T3SS; injectisome) is required for mycophagous ability, as the mutant bacterium is unable to forage over *R. solani*. Generally bacteria utilize the T3SS to deliver the effector proteins into its eukaryotic host and these effectors play crucial role during pathogenesis on plants as well as animals. By using computational tools, we analyzed the NGJ1 draft genome to identify potential T3SS effectors and observed a prophage tail-like protein (Bg_9562) in the list. Generally prophages are bacterial predators and upon induction they can kill bacterial cells. We observed that although the Bg_9562 gene is present in a bacteriophage cluster of NGJ1 but the cluster is incomplete as it lacks genes that encode head/capsid assembly proteins.

To further investigate, we obtained insertion mutants in the Bg_9562 gene and observed that the mutant bacterium is defective in mycophagous ability. Also the defect was fully complemented by expressing full length copy of the genes through broad host range plasmid. We further observed that during mycophagous interaction, the Bg_9562 protein gets delivered into fungal cells in a functional T3SS dependent manner. Also heterologous expression of the protein could impart mycophagous ability in *Ralstonia solanacearum*, an otherwise non-mycophagous bacterium. For gain of such ability the bacterium needs functional T3SS. Overall, our data suggested that the T3SS mediated delivery of a prophage tail-like protein (Bg_9562) into fungal cells is required for mycophagous ability.

**Figure 1:**
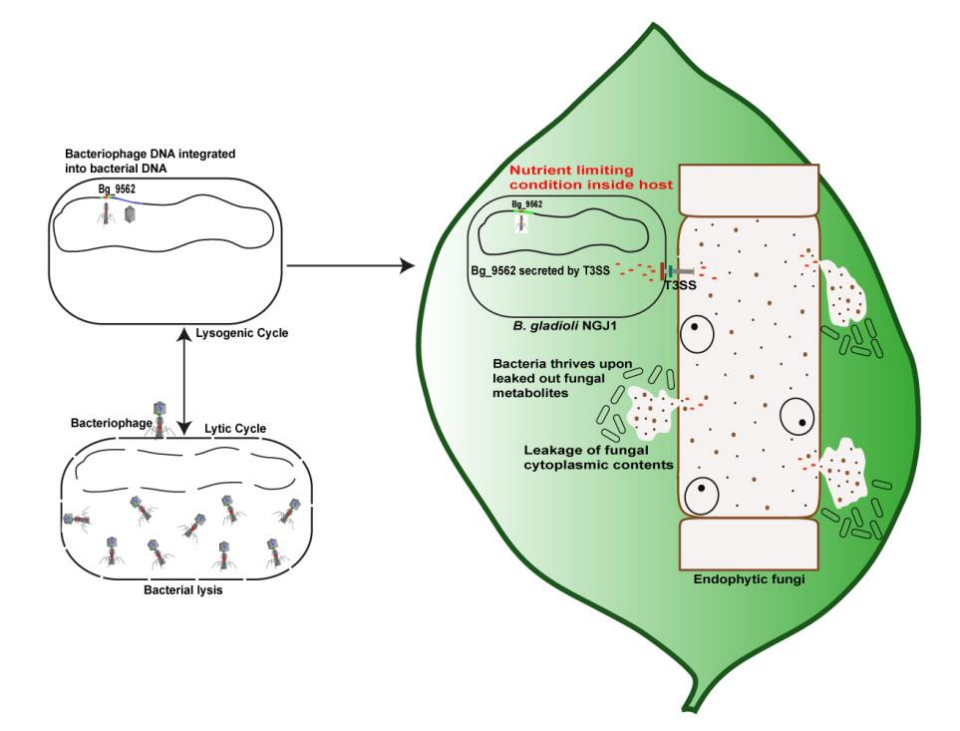
FIGURE 1: The mycophagous ability of NGJ1 would be advantageous for the bacterium to survive in a hostile environment. Bacterial genome contains bacteriophage clusters, which upon induction form phage particles and kill bacteria. However the *B. gladioli* strain NGJ1 had evolved to delete the head assembly proteins from the prophage cluster so that the phage particle cannot be induced. In this process, the bacterium seems to have evolved a T3SS signal in a prophage tail-like protein (Bg_9562) and delivers the protein into fungal cells in a functional T3SS dependent manner. By unknown mechanism, this induces fungal cell death responses and leads to disintegration of fungal hypha. This causes release of fungal cytoplasmic grannules as well as metabolites which the bacterium can utilize to sustain its growth. The mycophagous property of the bacterium can help it to survive under nutrient limiting condition. Considering this, we speculate the NGJ1 to be a hub bacterium which can reshape the entire plant associated microbiome and however this needs to be experimentally explored.

Generally it is difficult for the bacterium to survive in a hostile host plant environment. Bacteria share common habitat and have been co-evolving with fungi. The ability to feed on fungi, will be an advantageous trait for the bacterium to ensure availability of nutrients without harming the host plant. Our recent study suggests that during evolution the *B. gladioli* strain NGJ1 has adopted to utilize a phage tail-like protein, evolved a T3SS signal and deliver it into fungal cells by using T3SS (Figure 1). This triggers cell death and release of fungal metabolites which can be utilized by the bacterium as a nutrient source to sustain its growth. However it remains to be established how the prophage tail-like protein can induce cell death response. As we don’t observe any lytic or toxic domain associated with this protein, it is still intriguing how Bg_9562 proteinmight elicit cell death response pathway in fungi. Understanding of the signaling cascade and identifying interacting fungal protein(s) would further facilitate in unravelling the mystery associated with broad spectrum antifungal activity of Bg_9562, the prophage tail-like protein of *B. gladioli* strain NGJ1.

